# Short and long-term barriers and facilitators of skin self-examination among individuals diagnosed with melanoma

**DOI:** 10.1186/s12885-019-6476-5

**Published:** 2020-02-14

**Authors:** Adina Coroiu, Chelsea Moran, Catherine Bergeron, Martin Drapeau, Beatrice Wang, Abbas Kezouh, Jochen Ernst, Gerald Batist, Annett Körner

**Affiliations:** 1 Department of Social and Behavioral Sciences, Harvard T.H. Chan School of Public Health, Boston, MA USA; 2 Department of Educational and Counselling Psychology, McGill University, Montréal, Quebec, Canada; 3 Department of Psychology, University of Calgary, Calgary, Canada; 4 Department of Psychiatry, McGill University, Montréal, Canada; 5 Gerald Bronfman Department of Oncology, McGill University Health Center, Montréal, Canada; 6 Department of Epidemiology, Biostatistics, and Occupational Health, McGill University, Montréal, Canada; 7 Department of Medical Psychology and Medical Sociology, University of Leipzig, Leipzig, Germany; 8 Department of Medicine, McGill University, Montréal, Canada; 9 Department of Oncology, Sir Mortimer B. Davis-Jewish General Hospital, Montréal, Canada; 10 Department of Oncology, McGill University, Montréal, Canada; 11 Segal Cancer Centre, Montréal, Canada; 12 Centre for Translational Research in Cancer, McGill University, Montréal, Canada; 13 Lady Davis Institute for Medical Research, Jewish General Hospital, Montréal, Canada; 14 Louise Granofsky Psychosocial Oncology Program, Segal Cancer Center, Montreal, Canada; 15 Psychosocial Oncology Program, McGill University Health Centre, Montreal, Canada

**Keywords:** Skin self-examination, Melanoma early detection, Melanoma patients, Observational study

## Abstract

**Background:**

Melanoma can be lethal if not detected early and treated. Early detection can be facilitated via skin self-examination (SSE) and as such, SSE is part of melanoma follow-up care for individuals with a prior history, who face a life-long risk of reoccurrence. The objective of the current study was to identify short- and long-term predictors of SSE among melanoma survivors to inform future prevention interventions in high-risk groups.

**Method:**

This is an observational study with longitudinal assessments conducted with adult melanoma patients in active follow-up care.

**Primary outcome measures:**

Behavioral outcomes, comprehensive SSE (checking up to 5 body areas in the last 3 months) and optimal SSE (checking the entire body at least monthly in the last 3 months) were assessed at 3, 12, and 24 months post a dermatological educational session on skin cancer prevention. T tests and chi square analyses were used to examine changes in outcomes from 3 to 12 and 24 months. Linear and logistic regression models were used to examine the association between predictors and the primary outcomes.

**Results:**

Comprehensive SSE did not decrease significantly from 3 (M = 2.7, SD = 1.1) to 12 (M = 2.6, SD = 1.2) and 24 months (M = 2.4, SD = 1.2) post the education session, with the stronger predictor at all timepoints being intentions to perform SSE. Optimal SSE was higher at 3 months (59%) compared to 12 (46%) and 24 months (34%), with key predictors including self-efficacy and intentions to perform SSE and male sex at 3 months post; self-efficacy and reliance on medical advice at 12 months; and (lower) education and self-efficacy at 24 months.

**Conclusions:**

The key findings of this study are that 1) survivors maintain SSE behaviour over time, but rates of SSE performed in agreement with medical recommendations are higher immediately post standard dermatological education (i.e. usual care) and decrease somewhat over a 24-month period; and 2) the strongest psycho-social predictors of SSE are intentions and self-efficacy to perform the behavior, which are highly modifiable, for example via motivational interviewing and goal setting health interventions.

Melanoma is the 5th most common cancer in the United States [42] and the 7th in Canada [16]. The National Cancer Institute Surveillance, Epidemiology, and End Results (SEER) program projected that in 2019, there will be 96,480 new cases and 7230 deaths from melanoma in the US [42]. In Canada, of the total numbers of new cancer cases in 2015 (100,00 males, 96, 400 females), 3.6% in males and 3.2% in females were melanomas [15]. The Canadian Cancer Society estimates that in Canada there were 6800 new cases and 1150 deaths from melanoma in 2015. Melanoma is one of the most deadly human cancers and can metastasize when the primary tumor is only 1 mm in diameter, compared to most human cancers, which metastasize when they are 1 cm [10, 69]. Tumour thickness at diagnosis is the best predictor of survival [7–9, 29, 40]. Thus, early detection and timely treatment, i.e., surgical excision of the tumour are crucial to survival. The 5-year survival rates vary depending on stage at diagnosis and decline with more advanced staging at diagnosis: 95–100% stage I, 65–92.8% stage II, 41–71% stage III, and 9–28% stage IV [23]. A personal history of melanoma is associated with a life-long elevated risk for developing subsequent melanomas [14, 34, 73]. Melanoma survivors have a 9-fold increased risk to develop subsequent melanomas compared to the general population (range = 12.6 to 26.4-fold) and the risk remains elevated 20 years past the initial diagnosis [13, 74].

There is consensus within the clinical and scientific communities that 1) intervention strategies designed to reduce melanoma-related mortality must focus on early diagnosis of cancerous tumors [35, 63]; and that 2) intervention strategies will have the highest impact if targeting high-risk individuals [32, 34, 51, 63, 77]. Because some melanomas develop with a visible, pre-clinical phase, they can be amenable to early detection via visual inspection of the skin, by physicians and lay persons [30]. Evidence-based clinical guidelines for melanoma follow-up and care developed in the United Kingdom recommend that melanoma patients should be a) given a full body skin exam (with palpation of the lymph nodes) at every follow-up appointment, b) given information (written and verbal) about types of skin cancer and instructions on skin self-examination [56].

Clinical skin exams performed by physicians have been associated with thinner tumours at diagnosis [25, 26, 60, 70] and a 14% reduced risk of thick tumours, which frequently indicate advanced disease [3]. While clinical exams are undoubtedly useful for the early identification of cancerous skin lesions, most dermatological and cancer associations [5, 55];) as well as most clinical guidelines for the prevention of melanoma [50, 76] recommend that individuals at increased risk perform regular skin self-exams (SSE) in between medical follow-ups and present for medical skin exam if suspicious lesions are identified during skin self-exams.

## Skin self-examination (SSE)

There is evidence that the practice of SSE is beneficial for high-risk individuals. Empirical cross-sectional studies have found that patients and family members detected up to 50–80% of all melanomas [17, 18, 25, 70]. Also, increased thoroughness (or extent of skin covered) of the skin self-exam was associated with thinner lesions [60], and patients who examined at least some parts of their body had thinner lesions at diagnosis compared to those who did not examine their skin [17, 18, 60]. A case control study (423 melanoma patients and 678 matched controls) found that individuals who conducted SSE were twice as likely to self-detect melanoma and less likely to have thick (advanced) tumours at diagnosis compared to those who did not do SSE [72]. Finally, in a study with 1062 melanoma patients (stages I - II), among those who experienced a melanoma recurrence (19%), most recurrences were self-detected (55%) and led directly to seeking early medical advice [24]. Self-detection, not physician detection, independently predicted survival in this study.

Even though cutaneous melanomas are generally readily visible on the skin surface and individuals who perform SSE have a better prognosis than those who do not [4, 33, 44, 64, 71], most melanoma survivors do not perform whole-body skin exams regularly [12, 17, 18, 49, 53]. Reported SSE rates among melanoma survivors vary based on the definition of SSE used and the timeframe of assessment (Coroiu, Moran, Kwakkenbos, Thombs, & Körner: Preliminary evaluation of a melanoma knowledge questionnaire, in preparation). For example, a large cohort study conducted in Australia (*n* = 1433 confirmed melanoma cases) found that 57.4% of participants had performed an SSE in the past 3 years [58]. A cross-sectional study found that among 316 melanoma survivors, 28% reported having ever engaged in SSE, 16% reported doing monthly SSE, and 8% reported doing SSE every 2 months [38]. Another cross-sectional study (*n* = 321 melanoma patients) found that 15% of individuals performed skin checks by examining their moles every 1–2 months, 18% checked their skin every 6 months, and 17% checked their skin once a year [60]. Coups and colleagues [22] found that, although 72% of 176 melanoma patients had performed SSE during the last 2 months, only 14% had examined their whole body. These fluctuating rates of SSE reported in the literature illustrate a trend whereas only a small proportion of patients seem to be performing whole body SSE regularly, as per clinical recommendations; by missing certain body parts during the skin exam, opportunities for early detection of melanoma are also missed.

## Predictors of SSE

Personal characteristics that have been associated with SSE include a personal or family history of skin cancer, including melanoma [2, 36, 38, 64], being female, and having a higher level of education [17, 18, 38, 49, 58, 64]. Unlike medical and demographic factors linked to SSE, which are not generally amenable to intervention, psychosocial and educational factors associated with SSE are potential targets of interventions that aim to improve adherence to SSE instructions. Psychosocial factors associated with SSE behaviours in melanoma survivors and other high-risk individuals include greater knowledge about melanoma and SSE [27, 31, 64], higher perceived susceptibility to melanoma [6, 38, 58, 64], positive attitude towards SSE [64, 66], confidence in being able to perform an efficacious skin self-exam [6, 31, 64, 66], and having a physician recommend SSE [20, 49, 65, 66]. There is also preliminary evidence that cancer-related, occupational and financial distress were associated with increased frequency of SSE among melanoma survivors [45]. Furthermore, being informed about SSE by a health care professional has been shown to be associated with SSE performance [22, 48]. Some of the limitations of the literature exploring predictors of SSE include the lack of a standardized operationalization of SSE [21], which directly affects the reported rates of this behavior; the limited inclusion of psychosocial variables, such as distress, coping strategies and physician support, as only a few studies have addressed these constructs in relation to SSE; and limited duration of follow-up assessments, as research has shown that the performance of health behaviors decreases over longer time periods [28, 37].

In sum, there’s a strong argument from the empirical literature that the early detection of melanoma is associated with less advanced disease and as such with lower melanoma-related morbidity and mortality. While most melanomas are detected by patients, spouses, and other family members, physician-detected cancerous lesions tend to be thinner, representing an earlier disease stage, than self-detected lesions. Self-examination conducted in tandem with the clinical exam appears to be a desirable and more feasible approach to melanoma early detection than clinical exams alone. Despite SSE being an integral part of clinical guidelines for the prevention of melanoma among at-risk groups, many individuals at risk do not practice SSE regularly or thoroughly. Studies, including randomized controlled trials, have shown that rates of SSE can be improved through patient education, but little is known about those who adhere to SSE clinical recommendations versus those who do not. Acquiring knowledge on the strongest predictors of SSE practice will enable researchers and clinicians to design intervention protocols targeting core issues in melanoma prevention, and, thus, contribute to improved quality of life for patients, decreased need for invasive (but rarely curative) treatments such as chemotherapy, and ideally improved survival.

## Research objectives

The primary aim of this study was to identify short- and long-term predictors of SSE in a sample of patients with melanoma, who had been advised to perform SSE during a standardized dermatological education session on skin cancer prevention. In order to better understand the challenges and opportunities for secondary prevention of melanoma in this high-risk group, we had two specific objectives: (a) to assess rates of SSE behavior over time in a naturalistic setting, and (b) to identify individual-level predictors of SSE in the short (3 months) and long-term (12 and 24 months) following standardized recommendations for SSE. We anticipated that the self-reported rates of SSE behavior would be higher immediately post the dermatological education session compared to the rates reported at 12- and 24-month follow-ups. Hypothesized predictors of SSE in the short- and long-term included non-modifiable (i.e., biological sex, age, education, melanoma stage) and modifiable individual characteristics (i.e., knowledge about melanoma early detection, intentions to perform SSE, self-efficacy about SSE, physician support, psychological distress, and coping). We did not hypothesize a direction or magnitude for the associations between the non-modifiable predictors and SSE given that previous literature was inconclusive. We hypothesized that increased knowledge, intentions, and self-efficacy about performing SSE would be associated with higher rates of SSE in the short and long term, based on theoretical models of health behavior change that link these constructs to behavioral changes.

## Method

### Study design

This is an observational study with longitudinal follow-up (5 time points). We followed the STROBE guidelines for reporting of observational studies [75]. A detailed study protocol is available [46]. Personal characteristics (e.g., age, sex, education, ethnicity, years lived in Canada, marital status, mother tongue) were collected at enrolment (time 1). Disease-specific information (e.g., date of diagnosis, melanoma stage and depth) were collected from the patient medical charts and the pathology reports. A 20-min standardized educational session on skin cancer preventive behaviours modelled after best-practice guidelines of care for individuals at high-risk for melanoma was delivered within 3 to 6 months post enrolment (time 2). The hypothesized psychosocial predictors of SSE and SSE behaviour were assessed at 3 (time 3), 12 (time 4), and 24 (time 5) months post the educational session. Only participants who attended the educational session were retained in the longitudinal study and therefore included in the current analyses.

### Participants and procedures

Patients diagnosed with melanoma were recruited from Dermatology-Oncology Clinics of two McGill-affiliated hospitals in Montréal, Québec, Canada. Eligibility for the study included having a confirmed diagnosis of melanoma and receiving melanoma follow up care, being 18 years of age or older, and being proficient (written, verbal) in English of French. Recruitment was conducted in person by trained research assistants (RA), who explained the study procedures, assessed eligibility criteria, and obtained written consent. Consenting patients were offered the option to include their partners (spouses) in a dermatological educational session delivered at time point 2 (see below). Recruitment was conducted from September 2012 to March 2014 and data collection was completed in October 2016.

#### Ethics approval and consent to participate

Ethics approval for this study was granted by the Research Ethics Boards (REB) of the Faculty of Medicine, McGill University, the Jewish General Hospital (JGH) and the McGill University Health Center (MUHC). Written consent was obtained from all participants before study enrollment.

#### Dermatological educational session on SSE

The dermatological educational session, which was offered to all consenting participants and their partners, matched best-practice guidelines of care for individuals at high-risk for melanoma. It was delivered by three research assistants with backgrounds in medical sciences, nursing, and psychology, all of whom were trained by a dermatologist on the research team (BW). The educator briefly introduced herself, stated the purpose of the session (“how to best examine one’s skin for the early signs of melanoma”), explained the ABCDE criteria for the identification of problematic moles, illustrated how to systematically check the skin (checking all body parts with the help of another person), and provided take-home materials (e.g., body map diary, ABCDE book mark). A detailed document about the content of the educational session is available online at https://osf.io/ftw6v/?view_only=3eaa58a6f4654bd3af2064bbecbb15ad .

### Primary outcome measure: dependant variables

#### SSE behaviour

As there is no standardized method to assess SSE behaviour [21], we developed a scale based on items previously used in melanoma prevention research. We assessed SSE behaviour using 7 items, which inquired about the frequency of examining the skin on the entire body for problematic moles during the previous 3 months (e.g., “In the last 3 months, how often did you examine …”). The first 5 items inquired about the examination of separate body areas: 1) head and neck (face, neck and scalp), 2) front upper body (stomach, check, arms and shoulders), 3) front lower body (legs, genital/hip areas, top and bottom of feet, between toes), 4) back upper body (upper and lower back), 5) back lower body (buttocks and back of legs). Two additional items assessed whether participants had correctly examined the back areas, by using the help of another person (“In the last three months, how often did you have someone else help you with the skin self-exam”) or mirrors (“In the last three months, how often did you use a mirror for skin self-exams). Responses were scored on a 6-point scale: 0 (‘never’), 1 (‘once every 3 months’), 2 (‘once every 2 months’), 3 (‘once a month’), 4 (‘once a week’), 5 (‘more often’).

Scoring of the SSE Behavior Scale. To score the SSE behaviour scale, first we collapsed answer choices 4 (‘once a week’) and 5 (‘more often’) into one answer due to no prior evidence to suggest a benefit of “weekly” or “more frequent SSE” compared to “monthly SSE” (which is the recommended frequency for SSE, as per most clinical guidelines) and inconsistent distribution of scores for the “weekly” or “more often” categories in the current sample. Second, we compared the answers to the items inquiring about the scalp/neck and back areas (items 1, 4, 5) against the answers to the items inquiring about getting help during the exam from another person (item 6) or using mirrors (item 7) and adjusted the responses to the respective body areas to match the highest answer on either item 6 or 7. Our rationale for scoring the SSE behaviour variable in this manner was that in reality, participants could only adequately examine their backs if they received help from someone else or used two mirrors. Third, we used two separate conceptualizations of SSE in analyses: a) comprehensive SSE, i.e., the frequency of and the extent of skin covered during the skin self- exam, and b) optimal SSE, i.e., whole-body SSE performed at least monthly, as per our standardized patient education based on clinical care guidelines for individuals at increased risk.

#### Comprehensive SSE

Comprehensive SSE was operationalized as *frequency of examining up to 5 body areas in the last three months*. A sum score was computed across the 5 body part items (items 1–5), using corrected scores for the items asking about the back areas (3, 4, 5) against items 6 or 7, as per scoring methodology described above. Possible scores ranged from 0 to 20, where higher scores indicated more comprehensive SSE.

#### Optimal SSE

Optimal SSE was operationalized as *whole-body self-exam performed monthly or more often in the last 3 months*. To compute this variable, we used the 5 body part items with corrected scores for the items asking about the back areas, as per our scoring methodology described above, which we dichotomized into 1 = if all of the 5 body parts were checked at least monthly (“monthly “or “more often”) or 0 = if any of the 5 body parts were checked less often than monthly (“once every 3 months” or “once every 3 months”).

### Other measures: independent variables

#### Knowledge about melanoma early detection (Coroiu, Moran, Kwakkenbos, Thombs, & Körner: Preliminary evaluation of a melanoma knowledge questionnaire, in preparation).

Melanoma knowledge was assessed using a 6-item self-report measure covering melanoma risk factors and melanoma preventive behaviours (sample item, “Melanoma can develop a) on any skin surface; b) only on parts of the skin exposed to the sun”). The items were scored as “True” or “False”, with total sum scores computed across the 6 items ranging from 0 to 6. Validation analyses conducted in the current sample found that higher knowledge scores were associated with younger age and more positive attitudes about SSE, but was not associated with educational attainment, melanoma stage, or past SSE (Coroiu, Moran, Kwakkenbos, Thombs, & Körner: Preliminary evaluation of a melanoma knowledge questionnaire, in preparation).

#### Self-efficacy for SSE [11]

Self-confidence in performing effective SSE was measured using a 5-item self-report questionnaire. Response options ranged from 0 (“strongly disagree”) to 3 (“strongly agree”), with possible total scores ranging from 0 to 15 and higher total scores indicating higher levels of self-efficacy for SSE. Item 3 (“There are so many moles and freckles on my body that performing skin self-exams would be difficult”) was reverse coded. A previous investigation of the psychometric properties of the scale in data collected at enrolment found that it was reliable (α = 0.74) and positively associated with physician support and intentions to perform SSE [11].

#### Intentions to perform SSE [49]

Intentions to perform SSE were assessed using 1 item: “How likely are you to self-examine your skin on a regular basis in the coming year?”. The item was scored on a 5-point Likert scale ranging from 1 (“very unlikely”) to 5 (“very likely”), with higher scores indicating stronger intentions to perform SSE. In a study assessing intentions and adoption of SSE practice in patients with melanoma, higher intention to perform SSE was associated with female gender, physician recommendation of SSE, and patient perception of barriers and benefits of SSE [49].

#### Patient Health Questionnaire-4 (PHQ-4) [47]

The PHQ-4 is a 4-item scale assessing symptoms of depression (sample item, “Little interest or pleasure in doing things”) and anxiety (sample item, “Not being able to stop or control worrying”) over the past 2 weeks. The items are scored on a three-point scale, ranging from 0 (‘not at all’) to 3 (‘nearly every day’). Total scores range from 0 to 12, with higher scores indicating higher distress levels. In a sample of patients seeking treatment in primary-care settings, PHQ-4 scores were strongly associated with functional impairment and higher healthcare usage [47]. Higher PHQ-4 scores were also associated with longer duration of hospital stay, higher likelihood of re-hospitalization within 90 days and of death in patients with advanced cancer [57].

#### Skin Cancer index (SCI) [61]

Disease-specific emotional, social and appearance-related distress were assessed using a 15-item measure. This self-administered questionnaire asks about skin cancer worries in the past month (sample item, ‘During the past month, how much have you… felt anxious about your skin cancer’). Response options range from 1 (‘very much’) to 5 (‘not at all’), for a total possible sum score of 15–75. Items were reverse coded to improve comparability to other measures in the current study, where higher scores indicate higher levels of the measured construct. This scale demonstrated a high level of internal consistency (0.82 > α > 0.92) and good convergent and divergent validity among skin cancer patients [61], and was sensitive to detect changes in distress post-surgical treatment [62].

#### Reliance on medical advice (FKV-2)

The two-item subscale “compliance/ trust in doctor” of the Freiburg Questionnaire of Coping with Illness (FKV) [Freiburger Fragebogen zur Krankheitsverarbeitung] [54] was used to assess coping with melanoma by relying on medical advice (“I follow the medical advice exactly” and “I trust my doctors”). Response options ranged from 1 (‘not at all’) to 4 (‘very much’) with possible total scores between 2 and 8. Higher scores indicate more use of coping by adhering to medical advice. Items from this German scale were translated to English using forward-backward translation procedure [1, 19]. The total FKV scale (35 items) has shown good psychometric properties in a variety of samples with chronic illnesses [54] and the here used subscale had an acceptable internal consistency of 0.69 in a cancer sample [41].

#### Constructive attitudes about health (heiQ)

The 5-items constructive attitudes subscale of the Health Education Impact Questionnaire (heiQ) [59] was used to assess constructive attitudes and approaches to managing challenges of the cancer experience (sample item, “I try not to let my health problems stop me from enjoying life”). Response options range from 0 (‘strongly disagree’) to 3 (‘strongly agree’), with possible total scores between 0 and 15 and higher scores indicating increased attempts to minimize detrimental effects of illness upon one’s life. The heiQ questionnaire was originally developed to evaluate patient self-management and education programs [59]. Its adaptation to the cancer context was found to be reliable and valid in a large sample of Canadian cancer survivors, where sum scores of the constructive attitudes and approaches subscale were associated with self-efficacy and productive communication, and improved emotional and mental health [52].

### Data analysis plan

Descriptive statistics (means, standard deviations, percentages, percentiles) were computed for all study variables. Chi square and t tests were used to test changes in the two behavioral outcomes over time (3–12, 12–24, 3–24 months). Stepwise regression models, with all variables entered in one step were conducted with the two behavioural outcomes separately. Optimal SSE was modeled as a binary outcome and comprehensive SSE was modelled as a continuous outcome. The predictors were age, gender, education, melanoma stage, melanoma knowledge, SSE intentions, SSE self-efficacy, the skin cancer index (SCI), the PHQ-4, constructive attitudes and approaches subscale of the heiQ subscale, and over-reliance on medical advice (FKV-2). Given that we added a data collection (assessment) timepoint after the publication of the protocol and we experienced higher than anticipated attrition rates, we were not able to analyze the data, as per the plan in the protocol. We opted for descriptive, exploratory analyses as opposed to hypothesis-driven analysis of change in the outcome over time due to low sample size per time point. All analyses were computed using IBM SPSS v.21 [44].

## Results

### Study characteristics

**Fig. 1:**
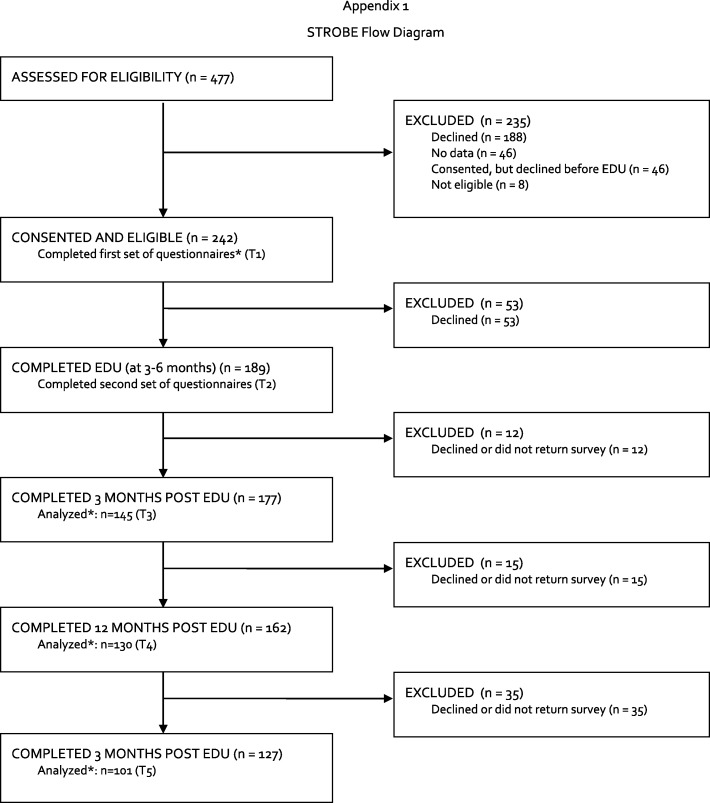
STROBE FLOW DIAGRAM. *Note.* EDU = standardized educational session. *The samples used in the current analyses included individuals at each assessment time point who provided complete data for all of the study measures

The participation flow chart was included in Figure 1 in Appendix. A total of 477 potentially eligible individuals were approached in person about participating in this study, 189 took part in the dermatological education session, 177 completed the first follow-up appointment (3 months post), 162 completed the second follow-up (12 months post) and 127 completed the third and last follow-up (24 months post). Data used in the current analyses pertains to the three follow-up time points and includes only participants, who provided complete data for all study measures: *n* = 145, *n* = 130, and *n* = 101. Table 1 includes sample characteristics and descriptive statistics: approximately half of our sample were females, the mean age was 60, the average number of years of education was 15, and roughly half of the sample had a stage I melanoma.

**Table 1:** Characteristics of the Study Sample, including Demographic Characteristics and Baseline Data for the Study Measures (*n* = 145)

Variable Name	Q1 (1^st^quartile)	Q2 (median)	Q3 (3^rd^quartile)	Mean (SD)	*N* (%)
Sex (F)					84.0 (51.2)
Age (in years)	51.0	60.0	68.8	59.2 (13.2)	
Education (in years)	12.0	15.0	17.0	14.9 (3.3)	
Melanoma stage					
0 (In situ)					27.0 (16.5)
1					83.0 (50.6)
2					36.0 (22.0)
3					10.0 (6.1)
4					5.0 (3.0)
Missing					3.0 (1.8)
Melanoma knowledge	0.7	0.8	1.0	0.8 (0.2)	
SSE self-efficacy	1.8	2.0	2.4	2.0 (0.5)	
SSE intentions	4.0	5.0	5	4.4 (0.9)	
SCI	1.4	1.8	2.4	2.0 (0.8)	
PHQ-4	0.0	0.3	0.9	0.5 (0.8)	
FKV-2	3.5	4.0	4.0	3.6 (0.5)	
heiQ	2.0	2.4	3.0	2.3 (0.6)	
Comprehensive SSE (Range 0–4)					
3-month (*n* = 145)	2.0	3.0	3.2	2.7 (1.1)	
12-month (*n* = 130)	1.8	3.0	3.4	2.6 (1.2)	
24-month (*n* = 101)	1.4	2.4	3.1	2.4 (1.2)	
Optimal SSE					
3-month (*n* = 145)					85.0 (58.6)
12-month (*n* = 130)					60.0 (46.2)
24-month (*n* = 101)					34.0 (33.7)

*Note*. *SSE* skin self-examination, *SCI* Skin Cancer Index, *PHQ-4* Patient Health Questionnaire-4, *FKV-2* 2 items of the Freiburg Questionnaire of Coping with Illness, assessing reliance on medical advice, *heiQ* The Health Education Impact Questionnaire, constructive attitudes about health subscale

### Rates and predictors of SSE behavior over time

#### Comprehensive SSE

The mean score for comprehensive SSE (defined as the frequency of checking of up to 5 body areas in the last three months; assessed on a scale of 0 to 4) decreased from 2.7 (3 months), to 2.6 (12 months) to 2.4 (24 months) post the dermatological session, as shown in Table 1. The changes in means from 3 to 12 months (*t*(273) = − 0.72, *p* = .45), 12–24 months (*t*(229) = − 1.26, *p* = .21), and 3–24 months (*t*(244) = − 2.03, *p* = .043) were minimal.

The final model predicting comprehensive SSE at 3 months post the dermatological education session included the following variables ranked by strength of association with the outcome: SSE intentions, male sex, SSE self-efficacy, higher melanoma stage, and reliance on medical advice (FKV-2); the model accounted for 38% of variance in SSE behavior whereby SSE intentions alone accounted for 27%. The final model predicting comprehensive SSE at 12 months post included SSE intentions, SSE self-efficacy, higher melanoma stage, and higher education; the model accounted for 30% of variance in SSE behavior whereby SSE intentions alone accounted for 21%. The final model predicting comprehensive SSE at 24 months post included SSE intentions, higher melanoma stage, higher education, and SSE self-efficacy; the model accounted for 44% of variance in SSE behavior whereby SSE intentions alone accounted for 23%. Age, knowledge about detection, distress (SCI and PHQ-4), and constructive attitudes about health (heiQ) were not related to comprehensive SSE. Detailed results per timepoint of assessment are included in Table 2.

**Table 2:** Stepwise Linear Regression Models Predicting Comprehensive SSE at 3^a^, 12^b^, and 24^c^ Months Post the Dermatological Education Session

Model	Variable^a^	R^2^	Δ R^2^	Δ F	β	p	Variable^b^	R^2^	Δ R^2^	Δ F	β	p	Variable^c^	R^2^	Δ R^2^	Δ F	β	p
1		.27	–	53.83		.000		.21	–	33.61		.000		.23	–	29.46		.000
	SSE intentions				.52	.000	SSE intentions				.46	.000	SSE intentions				.48	.000
2		.31	.04	7.68		.006		.25	.04	6.72		.011		.32	.09	13.18		.000
	SSE intentions				.54	.000	SSE intentions				.35	.000	SSE Intentions				.44	.000
	Sex (male)				.19	.006	Self-efficacy				.23	.011	Melanoma stage				.30	.000
3		.34	.03	6.79		.010		.27	.03	4.35		.039		.42	.09	15.55		.000
	SSE intentions				.45	.000	SSE intentions				.34	.000	SSE Intentions				.43	.000
	Sex (male)				.19	.008	SSE self-efficacy				.19	.028	Melanoma stage				.32	.000
	SSE self-efficacy				.20	.010	Melanoma stage				.16	.039	Education (in years)				−.31	.000
4		.36	.36	.02		.045		.30	.03	4.22		.042		.44	.03	4.60		.035
	SSE intentions				.43	.000	SSE intentions				.32	.000	SSE Intentions				.32	.001
	Sex (male)				.17	.014	SSE self-efficacy				.20	.020	Melanoma stage				.27	.001
	SSE self-efficacy				.18	.023	Melanoma stage				.16	.042	Education (in years)				−.29	.000
	Melanoma stage				.14	.045	Education (in years)				−.16	.042	SSE self-efficacy				.20	.000
5		.38	.02	3.93		.049	^b^Overall F (4, 129) = 13.18, *p* < .001						^c^Overall F (4, 100) = 18.95, *p* < .001					
	SSE intentions				.41	.000												
	Sex (male)				.15	.029												
	SSE self-efficacy				.17	.029												
	Melanoma stage				.15	.045												
	FKV-2				.14	.049												
	^a^Overall F (4, 144) = 16.95, *p* < .001																	

*Note*. *SSE* skin self-examination, *FKV-2* 2 items of the Freiburg Questionnaire of Coping with Illness, assessing reliance on medical advice

^a^Model predicting SSE behavior at 3 months. ^b^Model predicting SSE behavior at 12 months. ^c^Model predicting SSE behavior at 24 months post the dermatological education session

#### SSE optimal

The percentage of individuals who performed optimal SSE (defined as checking all 5 body parts at least monthly vs. less often than monthly in the last 3 months) decreased from 57% (3 months) to 44% (12 months) to 37% (24 months) post the dermatological session, as shown in Table 1. Changes in optimal SSE behavior from 3 to 12 months (χ^2^ (1) = 4.21, *p* = 0.04) and from 3 to 24 months (χ^2^ (1) = 14.72, *p* < 0.001) were statistically significant, but those from 12 to 24 months were not (χ^2^ (1) = 3.66, *p* = 0.055).

In analyses controlling for all variables, significant predictors of optimal SSE ranked by strength of association with the outcome at 3 months post the dermatological education session included SSE self-efficacy (OR = 15.31), SSE intentions (OR = 4.48, and female sex (OR = 0.32); at 12 months, significant predictors included reliance on medical advice (FKV-2; OR = 4.76) and SSE self-efficacy (OR = 2.95); and at 24 months, predictors included SSE self-efficacy (OR = 5.04) and (lower) education (OR = 0.69). Detailed statistics per timepoint are included in Table 3. Age, melanoma stage, knowledge about detection, distress (SCI and PHQ-4), and constructive attitudes about health (heiQ) were not related to optimal SSE.

**Table 3:** Stepwise Logistic Regression Models Predicting Optimal SSE at 3, 12, and 24-Months Post the Dermatological Education Session

	SSE at 3-months			SSE at 12 months			SSE at 24 months		
Variable	Optimal (*n* = 85)	Not Optimal (*n* = 60)	Adj* OR [95% CI]	Optimal (*n* = 60)	Not Optimal (*n* = 70)	Adj* OR [95% CI]	Optimal (*n* = 34)	Not Optimal (*n* = 67)	Adj* OR [95% CI]
Sex, Female	36.0 (42.4)	35.0 (58.3)	0.33 [0.12, 0.93]	28.0 (46.7)	37.0 (52.9)	1.00 [0.40, 2.52]	18.0 (52.9)	35.0 (52.2)	0.96 [0.22, 4.14]
Age	59.8 ± 12.6	59.0 ± 14.2	1.00 [0.96, 1.04]	60.2 ± 11.9	60.1 ± 15.5	1.00 [0.96,1.03]	58.6 ± 12.9	60.4 ± 13.5	0.94 [0.88, 1.00]
Education (in years)	14.6 ± 3.4	15.2 ± 3.4	1.01 [0.86, 1.14]	14.0 ± 3.2	15.3 ± 3.4	0.90 [0.79, 1.03]	13.5 ± 2.8	15.6 ± 3.3	0.69 [0.54, 0.88]
Stage 0 (in situ)	11.0 (12.9)	11.0 (18.3)	REF	6.0 (10.0)	13.0 (18.6)	REF	1.0 (2.9)	13.0 (19.4)	REF
1	44.0 (51.8)	34.0 (56.7)	0.51 [0.12, 2.08]	28.0 (46.7)	43.0 (61.4)	0.59 [0.15, 2.30]	18.0 (52.9)	39.0 (58.2)	NE
2	20.0 (23.5)	13.0 (21.7)	0.37 [0.08, 1.82]	20.0 (33.3)	12.0 (17.1)	1.85 [0.42, 8.20]	12.0 (35.3)	13.0 (19.4)	NE
3	5.0 (5.9)	2.0 (3.3)	0.78 [0.08, 7.72]	2.0 (3.3)	2.0 (2.9)	1.55 [0.13, 18.39]	1.0 (2.9)	2.0 (3.0)	NE
4	5.0 (5.9)	0.0 (0.0)	NE	4.0 (6.7)	0.0 (0.0)	NE	2.0 (5.9)	0.00 (0.00)	NE
Melanoma knowledge	0.8 ± 0.2	0.8 ± 0.2	0.36 [0.03, 4.93]	0.8 ± 0.2	0.8 ± 0.2	0.72 [0.08, 6.69]	0.8 ± 0.2	0.8 ± 0.2	3.59 [0.11, 118.01]
SSE intentions	4.8 ± 0.6	3.8 ± 1.0	4.48 [2.17, 9.23]	4.7 ± 0.7	4.1 ± 1.0	1.52 [0.81, 2.88]	4.9 ± 0.4	4.2 ± 1.0	4.26 [0.96, 18.88]
SSE self-efficacy	2.2 ± 0.4	1.8 ± 0.5	5.31 [1.53, 18.40]	2.2 ± 0.5	1.9 ± 0.5	2.95 [1.03, 8.43]	2.3 ± 0.4	1.9 ± 0.5	5.04 [1.11, 22.83]
SCI	2.0 ± 0.7	2.0 ± 0.8	0.80 [0.39, 1.62]	2.1 ± 0.8	1.9 ± 0.7	1.92 [0.95, 3.87]	2.2 ± 0.8	1.9 ± 0.7	3.03 [1.00, 9.24]
PHQ4	0.5 ± 0.8	0.6 ± 0.9	1.09 [0.51, 2.33]	0.4 ± 0.6	0.6 ± 0.9	0.75 [0.35, 1.59]	0.4 ± 0.7	0.5 ± 0.9	0.42 [0.11, 1.56]
FKV-2	3.7 ± 0.5	3.5 ± 0.4	2.54 [0.80, 8.03]	3.7 ± 0.5	3.5 ± 0.4	4.76 [1.48, 15.29]	3.8 ± 0.3	3.6 ± 0.4	3.06 [0.55, 17.05]
heiQ	2.4 ± 0.5	2.3 ± 0.6	0.55 [0.19, 1.54]	2.4 ± 0.5	2.3 ± 0.6	1.02 [0.38, 2.75]	2.4 ± 0.5	2.4 ± 0.6	1.00 [0.24, 4.22]

*Note*. Statistics for the SSE endorsed/not endorsed were presented as n (%) or M ± SD, where *M* mean and *SD* standard deviation, *NE* Not estimable

*Stepwise regressions adjusted for all of the study measures

*SSE* Skin self-examination, *SCI* Skin Cancer Index, assessing distress, *PHQ-4* Patient Health Questionnaire-4, assessing depression and anxiety, *FKV-2* 2 items of the Freiburg Questionnaire of Coping with Illness, assessing reliance on medical advice, *heiQ* The Health Education Impact Questionnaire, assessing constructive attitudes about health

## Discussion

To our knowledge, this is the first study in the literature to follow participants in melanoma follow-up care for as long as 24 month and periodically assess their self-surveillance (skin checking or SSE) behaviour in order to identify the key predictors of this behaviour under the condition of best-practice care in the context of secondary prevention of melanoma. The only other study to report a 24-month follow-up among melanoma patients is a randomized controlled trial testing the effect of delivering a melanoma prevention message via three different modalities against usual care on SSE conducted with a partner [67].

The first objective of this observational study with longitudinal follow-up was to assess the prevalence of SSE behaviours over time, i.e., SSE comprehensive and SSE optimal performance, following a standardized educational session on melanoma prevention and early detection via SSE. Comprehensive SSE was conceptualized as frequency and extent of skin covered during the skin exam while accounting for help while checking the back areas. SSE optimal was conceptualized as at least monthly whole-body exam and accounting for help while checking the back areas. The standardized educational session was designed to match the prevention strategies (information on early signs of melanoma and demonstration of how to perform SSE) recommended by dermatology associations and clinical care guidelines for patients in melanoma follow-up care. We found that comprehensive SSE remained stable from 3 to 12 and 24 months post the educational session, while optimal SSE decreased over time. Taken together, these results suggest that while individuals continued to examine their bodies for problematic moles to some extent throughout the study period, the percentage of those who adhered fully to the recommendations received during the educational session (and consequently to the recommendations included in the clinical guidelines of care for individuals with a prior history of melanoma) decreased from 3 to 12 months, but decreased less from 12 to 24 months. Of note, the reported rates of SSE behaviour found in this study are higher than those previously reported in cross-sectional studies [38, 58, 60]. This is potentially due to the fact that we offered all of our participants a standardized educational session on how to adequately examine one’s skin to identify problematic lesions, which may boosted everyone’s performance of SSE, some of which was maintained over time (see reported results for SSE comprehensive). Moreover, our repeated assessments may have acted as reminder or motivator for performing skin self-exams.

The second objective of this study was to assess the predictors of comprehensive and optimal SSE at 3, 12, and 24 months following a standardized educational session on SSE. Intention to perform SSE was the strongest predictor of comprehensive SSE in the short (3 months) and long-term (12 and 24 months). The strongest predictors of optimal SSE were self-efficacy and intentions for SSE in the short term (3 months) and self-efficacy for SSE in the long term (12 and 24 months). A possible explanation for these findings is that we used a high-risk sample in active follow-up by a dermatologist. Thus, our participants may have experienced higher levels of motivation (conceptualized as self-efficacy or confidence to perform a preventative behaviour *and* planning or intending to perform the behavior) to perform preventive behaviours, such as SSE, to begin with. It is also possible that our educational session boosted people’s motivation through the reinforcement of the benefits of such behaviour (e.g., early detection of melanoma). Further, at our recruiting hospitals, dermatologists routinely recommended lifelong SSE to all of their melanoma patients and occasionally demonstrated how to perform a skin exam, which might have contributed to elevated levels of self-efficacy for SSE throughout the study compared to patients followed by a general practitioner.

Other variables that showed a small association with increased comprehensiveness of the skin exam included male sex, increased self-efficacy, more advanced cancer stage, and increased reliance on medical advice at 3 months follow-up; and advanced stage, increased self-efficacy, and lower education level at 12 and 24 months follow-ups. Age, knowledge about detection, distress (general and melanoma-specific), and constructive attitudes about health were not related to either comprehensive nor optimal SSE. Of note, previous studies have found females, rather than males, to be more likely to perform SSE and higher levels of education to be associated with SSE [17, 18, 38, 49, 58, 64]. Given the low strength of the association between these variables and the SSE outcome in the current study, we recommend caution when interpreting these effects and their clinical relevance at this point.

## Limitations

There are some limitations of this study, which primarily relate to our sampling procedures, the study design, and the measurement of the behavioural outcomes. First, we aimed to include as many eligible participants as were seen at our recruitment’s centers during the active phase of the study. However, the skin cancer clinics were extremely busy, and some patients stayed on the premises strictly for the medical check-up, which made it difficult for us to approach them. It is possible that we missed some participants, who would have been eligible and could have provided valuable data to the study. Second, this is an observational study with longitudinal design, so we anticipated a 30% attrition rate. However, as the study extended to 24 months post the educational session, that is at least 27 months post enrolment, we experienced higher than expected loss to follow-up. For the current analyses we did not do imputations and chose to report results from study completers, which reduces our sample size considerably over time. Smaller numbers at subsequent time points affect the precision of the estimates reported and might affect the generalizability of our findings to other populations. In addition, it is possible that SSE behaviours were lower among individuals, who did not complete the study. Further, given that we did not collect data systematically on the number of dermatological appointments that occurred throughout the duration of our study, it is impossible to accurately assess the impact of clinical skin exams on the practice of skin self-exams. Furthermore, it is also practically impossible to speculate on the degree to which the clinical exam might have acted as a reminder or booster for participants’ own practice of SSE. Last, this study assessed the behavioral outcomes, comprehensive and optimal SSE via unvalidated self-report measures given that no standardized method of assessment currently exists. While we created the items based on items used previously in research and after consultation with experts, this might nonetheless affect the validity of our findings. Furthermore, recall bias might also play a role in how accurately SSE was reported.

### Clinical implications and directions for future research

The key findings of this study are 1) individuals at increased risk for melanoma, such as melanoma survivors in active follow-up care, maintain SSE behaviour over time, but rates of SSE performed in agreement with medical recommendations are higher immediately after the delivery of a brief standardized dermatological education on skin checking (recommendations for SSE and live demonstration) and tend to decrease somewhat over a 24-month period; and 2) the strongest psycho-social predictors of SSE are intentions and self-efficacy, which can be circumscribed to the larger construct of personal motivation to perform health behaviors. In order to boost adherence to recommended guidelines for SSE performance, future intervention studies with longer follow-up should include reminders and booster sessions. Long-term adherence to recommended guidelines (monthly whole-body SSE and timely seeking of medical opinion when problematic moles are identified) is crucial, as melanoma patients continue to experience an increased risk even 20 years past their diagnosis [13, 74].

To improve self-efficacy for the skin exam specifically, future intervention studies should include specific recommendations about how SSE should be performed, ideally accompanied by in-person demonstration of how to inspect the skin for early signs of melanoma, as well as concrete instructions about when to seek a medical opinion following a self-exam. While some of these recommendations have already been used and tested (e.g., [67]), future research is needed to optimize these recommendations and instructions for different at-risk populations.

To facilitate the setting of intentions to perform skin self-exams and subsequently ask for clinical exams, future studies could employ Motivational Interviewing strategies [68] to investigate personal desires and motivations (pro’s and con’s) to perform SSE, individual resources to support a long-term SSE practice (e.g., spousal support, knowledge of prevention and self-efficacy for SSE) and to address individual barriers, current and anticipated, to the adoption of skin cancer screening behaviors. In addition, interventions focused on effective goal setting, such as implementation intentions [39] could facilitate a long-term maintenance of SSE practice by creating individualized plans for “when”, “how”, and “where “to perform the SSE at different points in the future.

## Conclusion

To our knowledge, this is the first study in the literature addressing the short (3 months) and long-term (12 and 24 months) predictors of SSE behaviour in a sample of patients with melanoma in active follow-up. We found that the comprehensiveness of the skin exam did not decrease substantially over time and that its strongest predictor in the short and long term was the intention to perform the skin exam. We also found that optimal skin examination (or whole-body SSE performed at least monthly, as per recommendations) decreased over time. The strongest predictors of optimal skin examination were self-efficacy and intentions for the skin exam in the short term and self-efficacy and reliance on medical advice in the long term. These results have implications for the design of future melanoma prevention interventions, as intentions for SSE and self-efficacy for SSE are highly modifiable characteristics tapping personal motivation to perform preventative behaviours and can be targeted by psycho-social interventions (e.g., Motivational Interviewing and/or Implementation Intentions).

## Data Availability

The dataset generated and/or analysed during the current study are available in the Open Science Foundation repository, doi: 10.17605/OSF.IO/FTW6V [https://osf.io/ftw6v/?view_only=3eaa58a6f4654bd3af2064bbecbb15ad].

## Abbreviation

FKV-2: 2 items of the Freiburg questionnaire of coping with illness
heiQ: The health education impact questionnaire
PHQ-4: Patient health questionnaire-4
SCI: Skin cancer index
SSE: Skin self-examination
